# A transfer function based on bilinear transformation for narrow-band seismic record

**DOI:** 10.1371/journal.pone.0297999

**Published:** 2024-05-08

**Authors:** Zhiming Xu, Yashuang Bai

**Affiliations:** Institute of Civil Engineering, Southwest Forestry University, Kunming, China; Jamia Millia Islamia, INDIA

## Abstract

For a narrow-brand seismograph with a flat response range limited, it cannot precisely record the signal of a ground motion and output the records with the low-frequency components cut down. A transfer function is usually used to spread the spectrum of the narrow-brand seismic records. However, the accuracy of the commonly used transfer function is not high. The authors derive a new transfer function based on the Laplace transform, bilinear transform, and Nyquist sampling theory to solve this problem. And then, the derived transfer function is used to correct the narrow-band velocity records from the Hi-net. The corrected velocity records are compared with the velocities integrated from the synchronously recorded broad-band acceleration at the same station with Hi-net. Meanwhile, the corrected records are compared with those corrected by the Nakata transfer function. The results show that the calculation accuracy of the derived transfer function is higher than the Nakata transfer function. However, when the signal-to-noise ratio is below 24, its accuracy diminishes, and it is unable to recover signals within the 0.05–0.78Hz frequency band.

## 1 Introduction

Seismograph records, compared to acceleration records, offer a higher signal-to-noise ratio and more accurately represent true seismic motion. Displacement records derived from seismographs, through single integration, prove more suitable for multi-point excitation analysis than those from acceleration records, which are prone to algorithmic errors due to double integration. However, some signals in narrow-band seismograph records are suppressed, rendering them unsuitable for direct analysis. This paper aims to address signal distortion in narrow-band seismograph records and expand their application range.

A transfer function is the ratio of the Laplace transform (or z-transform) of the response (output) quantity of a linear system to the Laplace transform of the excitation (input) quantity under zero initial conditions [1]. It is a fundamental tool for analyzing and designing automatic control systems with a single input and single output signal.

As a complex domain model, the transfer function can process seismic records. Seismic records recorded by seismographs sometimes cannot reflect the real ground motion, due to the frequency characteristics limits of the instrument or the seismograph being affected by background noise [2,3]. These records combine real ground motion and noise. Researchers improve the seismic record’s signal-to-noise ratio [4–6] by deducting the seismograph’s response or determining the filtering order with the transfer function.

Additionally, the transfer function is used to correct narrow-band seismic records [7]. Zhang and Xu [8] applied the transfer function to correct components below 1 Hz in narrow-band seismic records, using velocity records as seismic excitation to calculate the earthquake response spectrum. Maeda et al. [9] developed a time-domain recursive filtering transfer function to repair narrow-band seismic records and recover distorted long-period ground motion signals. Nakata [10] used a transfer function expressed as zeros and poles to correct the Hi-net narrow-band velocity record, improving the amplitude and phase of ground motion.

These research efforts have enhanced the transfer function’s calculation efficiency and expanded the records’ frequency range. However, an in-depth study on the calculation accuracy of the transfer function is lacking. The pulse response invariance method, commonly used to establish the transfer function [11], yields fewer coefficients and amplifies low-frequency amplitude, leading to frequency aliasing and reduced accuracy. Researchers have explored factors affecting the transfer function’s calculation accuracy from the selection, aging, temperature compensation, and other aspects of the electronic components of the seismograph [12], but no specific measures to improve accuracy were presented.

Thus, we derived a new transfer function based on bilinear transformation, Laplace transformation, and Nyquist sampling theory, using Hi-net’s narrow-band seismic records to test its accuracy. This function, along with the Nakata transfer function, is used to correct Hi-net’s narrow-band seismic records, highlighting the differences among records corrected by these functions. This research offers a new transfer function for recovering suppressed long-period signals, converting narrow-band seismic records into broad-band seismic records.

## 2 Transfer function derivation

The relationship between the input signal and the output signal of a seismograph is as follows:

$$\ddot{x}(t)+2\xi \omega \dot{x}(t)+\omega^{2}x(t)=\ddot{y}(t)$$ (1)

where, *ω* is the natural angular frequency, *ξ* is the damping constant, *x*(*t*) is the output displacement of the seismograph, $\dot{x}(t)$ is the output velocity of the seismometer, $\ddot{x}(t)$ is the output acceleration of the seismometer, and $\ddot{y}(t)$ is the input ground motion acceleration.

The response transfer function of the seismograph as complex frequency is [13]:

$$H(\omega )=c\frac{(i\omega -z_{1})(i\omega -z_{2})(i\omega -z_{3})\ldots }{(i\omega -p_{1})(i\omega -p_{2})(i\omega -p_{3})\ldots }$$ (2)

In the formula, *c* is the scale factor, which is usually 1; the root of the numerator polynomial is called the zero-point z, and the root of the denominator polynomial is called the pole p, and the pole is determined by the natural frequency and damping ratio of the seismograph.

Using Eqs (1) and (2), the displacement transfer function can be obtained:

$$H_{d}(\omega )=\frac{\left(i\omega -z_{0})(i\omega -z_{1})(i\omega -z_{2}\right)}{\left(i\omega -p_{1})(i\omega -p_{2}\right)}$$ (3)

The velocity transfer function can be obtained, for Eq (3) over *iω*:

$$H_{v}(\omega )=\frac{\left(i\omega -z_{0})(i\omega -z_{1}\right)}{\left(i\omega -p_{1})(i\omega -p_{2}\right)}$$ (4)

Where *z*_0_ and *z*_1_ represent the zero of the transfer function, both of which are (0,0), and p_1_ and p_2_ represent the two poles of the transfer function. The formula for solving the poles is:

$$\begin{array}{c} p_{1}=-\omega_{0}(h+\sqrt{h^{2}-1})\\ p_{2}=-\omega_{0}(h-\sqrt{h^{2}-1})\\ p_{1}+p_{2}=-2\omega_{0}h\\ p_{1}*p_{2}=\omega_{0}^{2} \end{array}$$ (5)

In the formula, *ω*_0_ is the natural frequency, and *h* is the damping ratio.

When *iω* is represented by using a complex variable *s*, the transfer function of the seismograph in the Laplace domain can be expressed as:

$$H(s)=\frac{s^{2}}{s^{2}+2\omega_{0}hs+\omega_{0}^{2}}$$ (6)

Since the frequency range of Eq (6) has no restriction. The *ω* contains some frequencies higher than the Nyquist frequency, which will cause spectrum aliasing. Therefore, compressing the s-domain between $\left(-\frac{\pi }{T},\frac{\pi }{T}\right)$ by the tangent relationship is needed, where T is the sampling interval.

The conversion relationship is:

$$\omega_{1}=\frac{2}{T}\tan(\frac{\omega T}{2})$$ (7)

Using the mapping between the s plane and z plane $z=e^{i\omega_{1}T}$ [14,15], the bilinear transformation formula is obtained [16–18]:

$$s=\frac{2}{T}\frac{1-z^{-1}}{1+z^{-1}}$$ (8)

Substituting Eq (8) into Eq (6), it can be obtained:

$$\begin{array}{c} H(s)=\frac{4(1-2z^{-1}+z^{-2})}{az^{-2}+bz^{-1}+c}\\ a=4-4T\omega_{0}h_{0}+\omega_{0}^{2}T^{2}\\ b=2\omega_{0}^{2}T^{2}-8\\ c=4+4T\omega_{0}h_{0}+\omega_{0}^{2}T^{2} \end{array}$$ (9)

After that, the frequency range of Formula (7) is applied to Formula (9), and the coefficients in Formula (9) are obtained:

$$\begin{array}{c} a=1-2h\;\tan(\frac{\omega T}{2})+\tan^{2}(\frac{\omega T}{2})\\ b=2\tan^{2}(\frac{\omega T}{2})-2\\ c=1+2h\;\tan(\frac{\omega T}{2})+\tan^{2}(\frac{\omega T}{2}) \end{array}$$ (10)

The above formula is used to get the transfer function for a broad-band seismograph and a narrow-band seismograph. The two transfer functions’ coefficients calculated according to Formula (10) are used to get the biquadratic transfer function for correcting the narrow-band velocity record, as follows:

$$H(s)=\frac{H_{OB}(z)}{H_{OH}(z)}$$ (11)

H_OB_(z) and H_OH_(z) represent the transfer function of the narrow-band seismograph and the transfer function of the broad-band seismograph.

The above derivation process shows that the Laplace transform converts the seismograph’s natural frequency *ω* into complex frequency *s*. Since *s* has the advantage of describing repetition frequency, the growth rate, and the decay rate of the oscillation amplitude, the Laplace transform solves the defect that the Fourier transform’s natural frequency *ω* can only describe repetition frequency. Therefore, compared with the transfer function based on the Fourier transform, the transfer function based on the Laplace transform can reflect seismic signal variations more accurately.

The Nyquist sampling theory and bilinear transformation are applied while solving the transfer function. The former can limit the sampling frequency range and eliminate the spectral aliasing caused by the multi-valued mapping between the s-domain and the z-domain. The latter can realize obtaining the biquadratic transfer function in the z-domain.

## 3 Verification of transform function

The Hi-net employs narrow-band seismometers, and at some stations, both KiK-net broad-band strong motion seismographs and STS-2 broad-band seismographs (F-net) are installed [19]. The seismic records from Hi-net, KiK-net, and F-net are narrow-band, broad-band, and broad-band respectively, and are openly accessible.

Therefore, this paper selects Hi-net’s narrow-band velocity records to verify the accuracy of the newly derived transform function. The ground motions used are listed in Table 1. The flat response range of the Hi-net seismograph is limited to 1–20 Hz, leading to suppression of frequency components below 1 Hz and consequent distortion of the low-frequency components in the records.

**Table 1:** Ground motions.

Number	Hi-net /KiK-net	Earthquake name	Earthquake time (JST)	Epicenter latitude and longitude	Earthquake depth(km)	Magnitude/ Direction	Hi-net Signal-to-noise ratio	KiK-net Signal-to-noise ratio
1	N. TSKH/IBRH19	Ibaraki earthquake	20080508 01:45:00	36.227°N 141.607°E	51	M7.0/EW	72.45	48.55
2	N. TSKH/IBRH19	Chiba earthquake	20110319 18.56.43	36.21N 140.09E	76	M6.1/UD	37.94	20.03
3	N. TSKH/IBRH19	Miyagi earthquake	20110407 03:54:00	38.20°N 141.92°E	66	M7.1/EW	23.97	12.10
4	N. TSKH/IBRH19	Iwaki earthquake	20110731 03:54:00	36.90°N 141.22°E	57	M6.5/EW	102.16	52.60
5	N. TSKH/IBRH19	Fukushima earthquake	20210213 08:00:00	37.73°N 141.70°E	55	M7.3/EW	85.30	55.51
6	N-TSKH/IBRH10	Chiba earthquake	20121207 17:19:18	38.018°N 143.867°E	49	M7.3/EW	112.42	85.09
7	N-TSKH/IBRH10	Ibaraki earthquake	20161122 06:00:16	37.353°N 141.603°E	25	M7.4/EW	171.34	81.80

The coefficients in Formula (11) are calculated using the natural frequency *ω* = 2*π*(*Hz*) and damping ratio *h* = 0.7 of the narrow-band seismometer (Hi-net) and the natural frequency *ω* = 1/135(*Hz*) and damping ratio *h* = 0.707 of the broad-band seismometer (F-net). The coefficients are shown in Table 2.

**Table 2:** Coefficient of transfer function.

	a_i_	b_i_	c_i_
i = 1	1.044984	−1.998024	0.956990
i = 2	1.000370	−1.999999	0.999629

**Fig 1:**
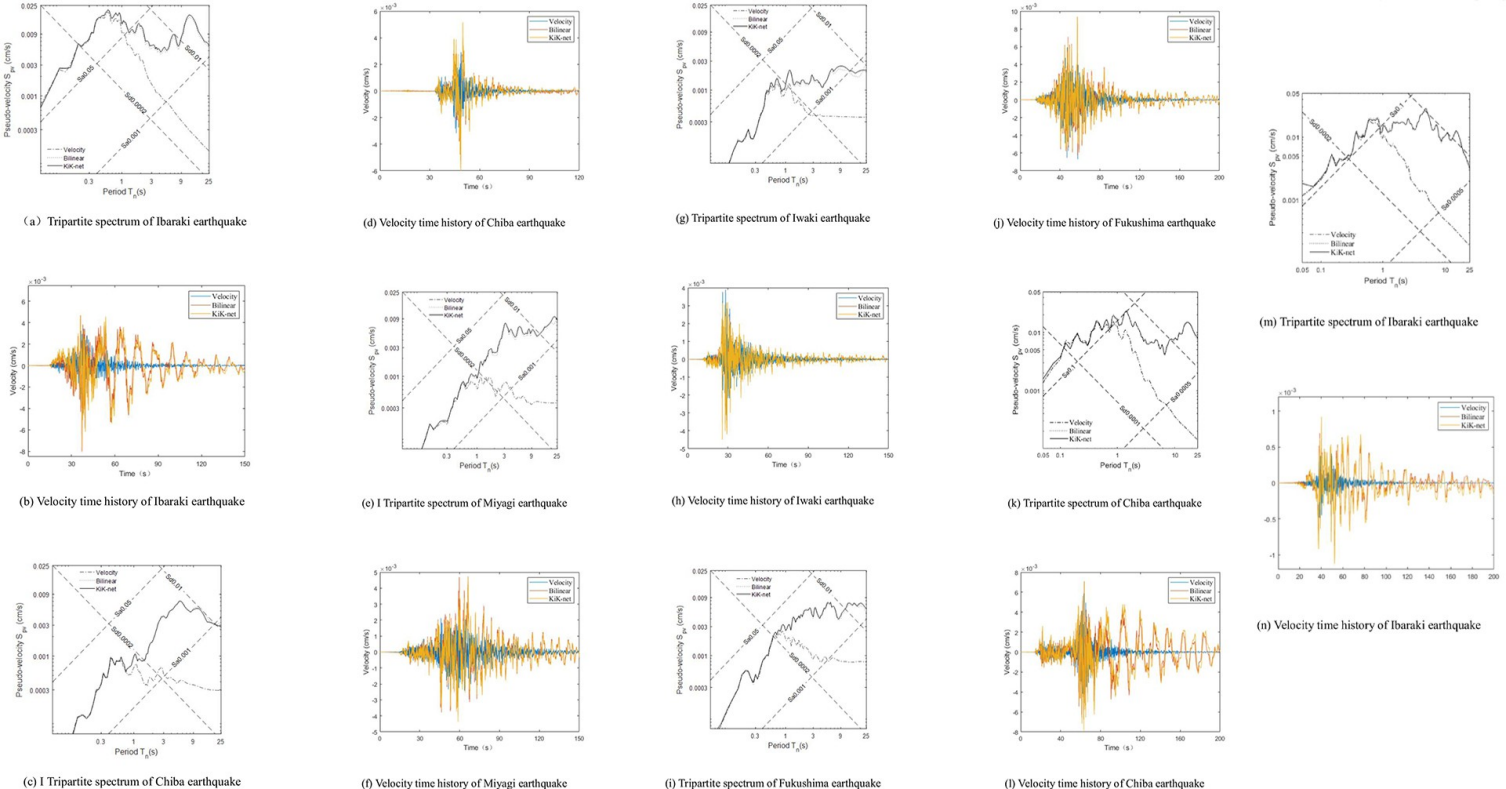
Spectrum and velocity time history used in the analysis. (a) Tripartite spectrum of Ibaraki earthquake. (b) Velocity time history of Ibaraki earthquake. (c) Tripartite spectrum of Chiba earthquake. (d) Velocity time history of Chiba earthquake. (e) Tripartite spectrum of Miyagi earthquake. (f) Velocity time history of Miyagi earthquake. (g) Tripartite spectrum of Iwaki earthquake. (h) Velocity time history of Iwaki earthquake. (i) Tripartite spectrum of Fukushima earthquake. (j) Velocity time history of Fukushima earthquake. (k) Tripartite spectrum of Chiba earthquake. (l) Velocity time history of Chiba earthquake. (m) Tripartite spectrum of Ibaraki earthquake. (n) Velocity time history of Ibaraki earthquake.

The tripartite spectra and velocity time histories of the five ground motions are depicted in Fig 1. These curves represent the KiK-net (velocity integrated from KiK-net’s acceleration), Bilinear (Hi-net’s velocity corrected by the derived transfer function), and Hi-net (the original Hi-net’s velocity).

The earthquake response spectra indicate that the three curves overlap within the 0–1 second range. Beyond 1 second, the discrepancies between the Hi-net and the standard grow with increasing period. The differences between the Bilinear and the standard remain minimal, not exceeding 5% and increasing after 20 seconds but not surpassing 15%. The velocity time history reveals a lack of coincidence between Hi-net and the standard, whereas the Bilinear and the standard display identical waveforms. This suggests that the long-period component in Hi-net’s narrow-band velocity record is distorted, yet the distortion is effectively corrected by the derived transfer function, aligning closely with the standard.

## 4 Sensitivity of the parameters in the derived transfer function

In Formula (9), the transfer function’s six parameters relate only to the damping ratio and natural frequency of the narrow-band and broad-band seismograph [20,21]. These parameters have functional relationships and can be grouped into two categories. When using this transfer function, the rounding of significant digits in the parameters and slight fluctuations in the instrument parameters of seismographs may impact these six parameters.

In the sensitivity analysis, assuming the existing results are accurate, the damping and natural frequency of seismographs are adjusted according to their respective deviations of 10%, both deviations of 10%, and the number of significant digits of the parameters reduced to 7, respectively.

Table 3 shows the differences in peak velocity of the five ground motion records. The Ibaraki earthquake record exhibits notable differences, with error rates of 5.13%, 4.49%, 1.71%, and 16.88%.

**Table 3:** Comparison of peak value with parameter errors (10^−3^ cm•s^-1^).

Number	Hi-net /KiK-net	Earthquake name	Exact value	Frequency 10%	Damping 10%	Common deviation 10%	7 significant digits
1	N. TSKH/IBRH19	Ibaraki earthquake	4.68	4.92	4.89	4.60	5.47
2	N. TSKH/IBRH19	Chiba earthquake	4.73	4.14	4.75	4.03	4.69
3	N. TSKH/IBRH19	Miyagi earthquake	5.17	4.66	5.05	4.51	5.18
4	N. TSKH/IBRH19	Iwaki earthquake	3.17	3.30	3.02	3.21	3.15
5	N. TSKH/IBRH19	Fukushima earthquake	9.37	8.66	8.76	8.27	9.35

**Fig 2:**
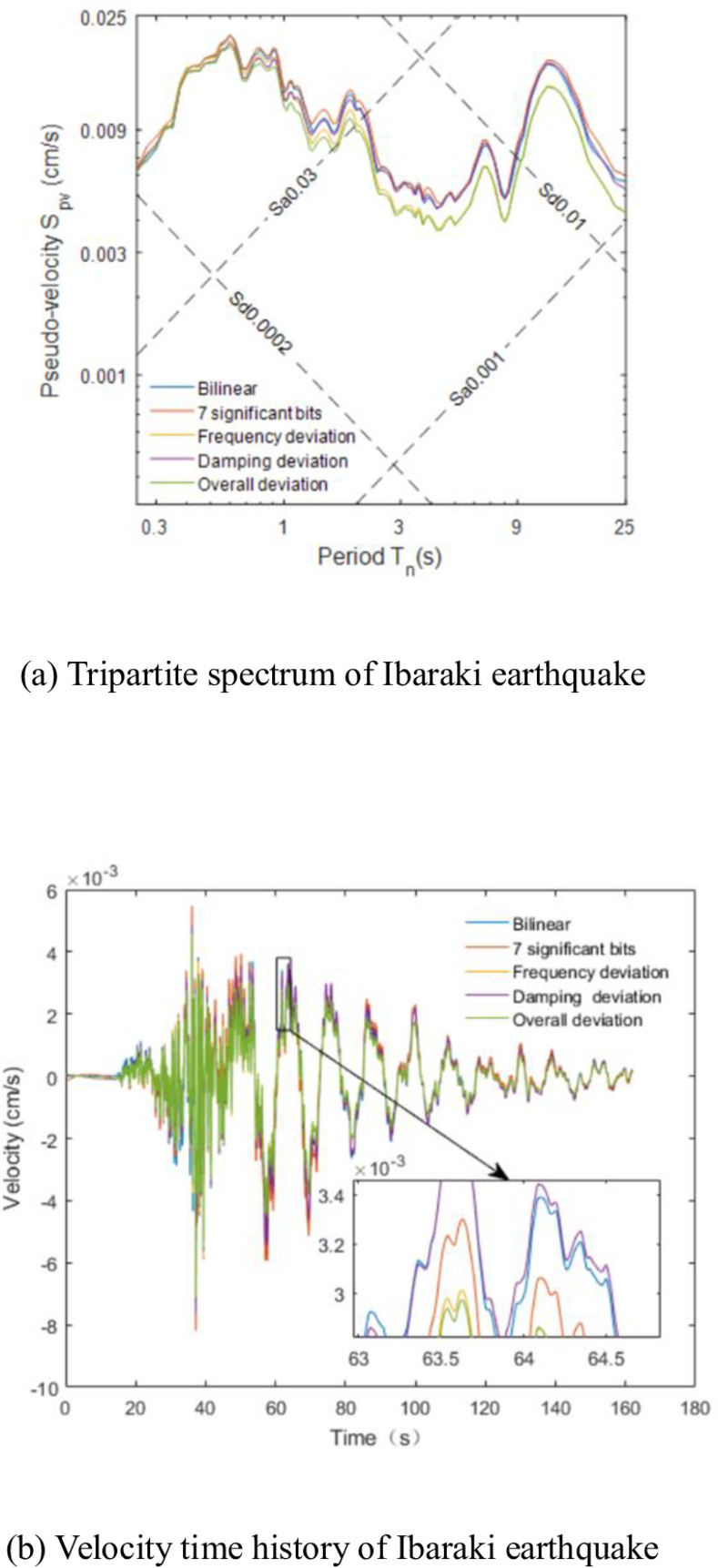
Comparison of parameter errors. (a) Tripartite spectrum of Ibaraki earthquake. (b) Velocity time history of Ibaraki earthquake.

The tripartite spectrum and velocity time history of the Ibaraki earthquake are presented in Fig 2. The figure illustrates significant differences in the tripartite spectra over a long period, although not exceeding 5%; the velocity time history curves are coincident. The errors resulting from these four deviations fall within an acceptable range, demonstrating the derived transfer function’s stability.

## 5 Comparison and discussion

The narrow-band seismometer in Hi-net does not cover the 0-1Hz range. To address this, Nakata proposed a transfer function for narrow-band velocity records to reconstruct signals in this frequency band. The transfer function is influenced by the narrow-band seismograph’s parameters, which define its zeros and poles. The Nakata transfer function is:

$$H_{a}(t)=\int_{-\infty }^{\infty }\frac{\left(i\omega -p_{0})(i\omega -p_{1}\right)}{i\omega -z_{0}}H_{v}(\omega )e^{-i\omega t}d\omega$$ (12)

Where *H*_*a*_(*t*) is the corrected time-domain acceleration record, and *H*_*v*_(*t*) is the original Hi-net velocity record.

**Fig 3:**
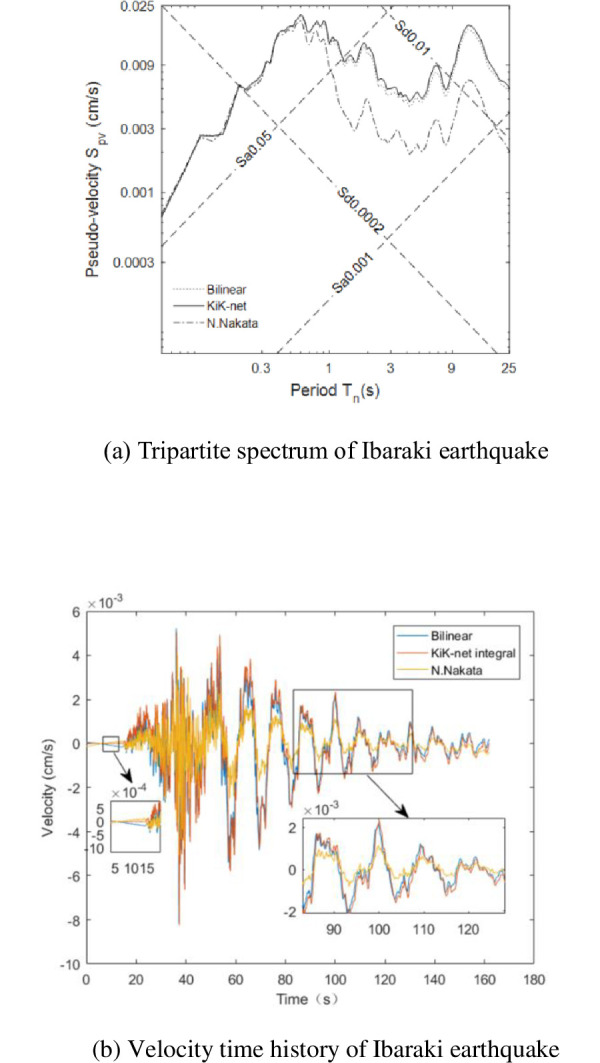
Comparison of corrected results of Ibaraki Prefecture by derived transfer function and N. Nakata transfer function. (a) Tripartite spectrum of Ibaraki earthquake (b) Velocity time history of Ibaraki earthquake.

**Fig 4:**
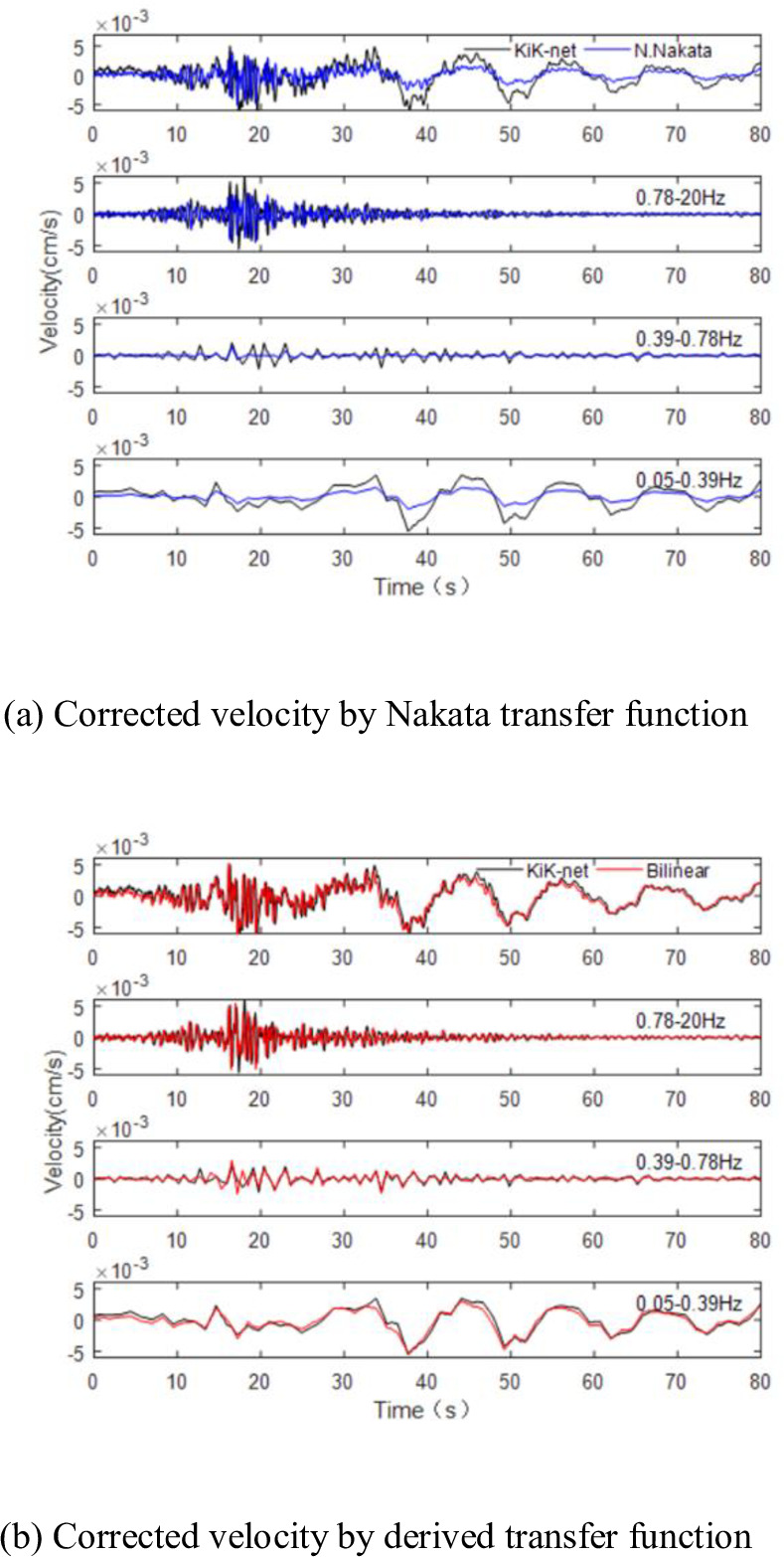
Comparison of the Ibaraki earthquake in different frequency bands. (a) Corrected velocity by Nakata transfer function. (b) Corrected velocity by derived transfer function.

The velocity records in Table 4 have been corrected using the Nakata transfer function and compared with those corrected by the derived transfer function. The corrected results of the five ground motion records are consistent. Therefore, only the tripartite spectrum and velocity time history for Ibaraki Prefecture are provided (Fig 3). In the figure, the ’Nakata’ curve represents the velocities corrected by the Nakata transfer function. Fig 4 displays the corrected velocities, segmented by wavelets in different frequency bands, with the black line for KiK-net, red line for Bilinear, and blue line for Nakata.

**Table 4:** Comparison of the peak velocity.

Number	Hi-net /KiK-net	Earthquake name	Hi-net record	Corrected by Nakata transfer function	Corrected by derived transfer function	KiK-net integral velocity
1	N. TSKH/IBRH19	Ibaraki earthquake	3.4	4.0	4.7	5.0
2	N. TSKH/IBRH19	Chiba earthquake	2.9	3.0	5.2	5.0
3	N. TSKH/IBRH19	Miyagi earthquake	2.1	2.8	4.7	5.4
4	N. TSKH/IBRH19	Iwaki earthquake	2.3	2.4	3.1	2.7
5	N. TSKH/IBRH19	Fukushima earthquake	5.9	6.5	9.4	10.2

The tripartite spectral distribution and the velocity-time histories from the KiK-net are consistently utilized as benchmarks. The velocity-time history, as adjusted by the applied transfer function, aligns with the standard illustrated in Fig 3. In contrast, the velocity-time history modified using the Nakata transfer function exhibits a ’ringing’ effect in the foreshock region. It matches the standard in the main shock region but is noticeably less than the standard post-main shock. The tripartite spectra, when corrected with these two functions, show a resemblance to the established standard.

The frequency band analysis, it is observed that within the 0.05–0.78Hz range, both the contour and peak value of the velocity, corrected using the derived transfer function, are in congruence with the standard. However, the peak value of the velocity, as corrected by the Nakata transfer function, falls below the standard. In the higher frequency range of 0.78-20Hz, the velocities adjusted by both functions display similarity to the standard.

In summary, the velocity records corrected by the derived transfer function are more accurately matched in terms of velocity time history, peak value, and spectrum characteristics, effectively extending the narrow-band record’s usable frequency range to 0.05-20Hz.

## 6 Conclusion

The transfer function is primarily utilized for restoring ground motion or simulating velocity, acceleration, and displacement within certain frequency bands. This study expands its application, deriving a transfer function based on the Laplace transform, bilinear transform, and Nyquist sampling theory for spreading narrow-band velocity records.

Seven Hi-net narrow-band velocity records, corrected using this derived function, align with velocities integrated from broad-band acceleration recorded at the same Hi-net stations.

The analysis indicates that with a signal-to-noise ratio above 24, the derived transfer function reliably expands the effective frequency range of narrow-band velocity recordings to 0.05-20Hz.

The Nakata transfer function, while correcting Hi-net’s narrow-band velocity records, fails to recover suppressed signals within the 0.05–0.78Hz band.

This research lays a theoretical foundation for enhancing the calculation accuracy of the transfer function and broadens the application range of narrow-band velocity records.

## Supporting information

S1 File Supplement data about the drawings in this manuscript. (RAR)

S2 File Contains the complete set of codes used for creating the figures presented in this paper using MATLAB software. (RAR)
